# Global and regional burden of pneumoconiosis, 1990–2021: an analysis of data from the global burden of disease study 2021

**DOI:** 10.3389/fmed.2025.1559540

**Published:** 2025-03-28

**Authors:** Ye Chen, Dandan Liu, Huixia Ji, Wenying Li, Yuhua Tang

**Affiliations:** Department of Occupational Disease, Nanjing Prevention and Treatment Center for Occupational Diseases, Jiangsu, China

**Keywords:** pneumoconiosis, global burden of disease, socio-demographic index, incidence, death

## Abstract

**Background:**

Pneumoconiosis remains a widespread occupational disease globally. This study provides an updated overview of the global burden of pneumoconiosis, examining incidence and mortality from 1990 to 2021.

**Methods:**

The study assessed the incidence and mortality of pneumoconiosis using GBD data from 1990 to 2021, presenting findings as point estimates with 95% uncertainty intervals.

**Results:**

In 2021, there were 62,866 new pneumoconiosis cases and 18,323 deaths worldwide, with age-standardized incidence (ASIR) and age-standardized mortality rates (ASMR) of 0.736 and 0.219 per 100,000 population, respectively, showing decreases of 28.5 and 52.8% since 1990. The highest ASIR and ASMR of pneumoconiosis were found in middle and high-middle SDI quintiles in 2021, with East Asia having the highest ASIRs. ASIR and ASMR generally declined but rose in Australasia and Oceania from 1990 to 2021. Globally, the number of incidences peaked at 65–69 years in 1990 and 2021, with death peaks at 65–69 years in 1990 and 80–84 years in 2021. A correlation analysis revealed that ASIR and ASMR either decreased or remained stable in the majority of countries and territories as SDI increased. Decomposition analysis shows that population growth and aging drove the global number increase in most regions, while epidemiological changes had a negative impact.

**Conclusion:**

Despite an overall decline in global pneumoconiosis burden from 1990 to 2021, particularly in the middle and high-middle SDI quintiles, and in East Asia. The future looks promising, but pneumoconiosis remains a public health concern. The implementation of prevention strategies and improving the quality of life of current patients should be a priority.

## Introduction

Pneumoconiosis is a chronic pulmonary disease characterized by diffuse fibrosis of lung tissue due to prolonged occupational exposure to dust (1). Coughing, sputum production, chest tightness, and dyspnea are common symptoms. It can result in progressive pulmonary fibrosis, pulmonary arterial hypertension, cor pulmonale, and right heart failure, severely impairing patients’ quality of life and imposing a substantial economic burden on the community. Disease burden varies markedly across regions and socioeconomic groups due to disparities in occupational health standards and resources (2, 3).

Silicosis accounts for a predominant proportion, approximately 90%, of all diagnosed pneumoconiosis cases. The epidemiological data on silicosis from the Global Burden of Disease (GBD) for 2019 indicated an increase in both the number of incidence and mortality among patients (3). However, following age standardization, both indicators exhibited a downward trend. The burden of pneumoconiosis has evolved globally over time. It is essential to establish consistent, comparable, and systematic analyses of the disease burden and trends of pneumoconiosis in different regions of the world in order to formulate effective intervention strategies.

The present study offers updated findings from the GBD regarding pneumoconiosis for the year 2021. In this study, the incidence and deaths of pneumoconiosis, socio-demographic index (SDI), as well as the potential causes of these epidemiological changes in GBD from 1990 to 2021, were systematically visualized for ages, years, and regions worldwide. As a consequence of the availability of estimates regarding the disease burden associated with pneumoconiosis, clinicians, epidemiologists, and health policymakers will be better positioned to optimize the allocation of medical resources and devise more effective public health interventions.

## Methods

### Data sources

In this study, the data on pneumoconiosis were obtained from the GBD 2021, which offers the most current epidemiological estimates regarding the burden of 459 diseases and injuries across 204 countries and territories worldwide, spanning 1990 to 2021. The data for this analysis are publicly available via the Global Health Data Exchange^1^ (4). Detailed descriptions of the data, methods, and statistical approaches can be found in previous publications (5, 6). Moreover, the Institutional Review Board at the University of Washington exempted the need for informed consent for accessing GBD data (7). The study adhered to the Guidelines for Accurate and Transparent Health Estimates Reporting (8).

The GBD study utilized an advanced modeling approach to quantify the burden of pneumoconiosis. DisMod-MR 2.1, a Bayesian meta-regression disease modeling tool, was used to calculate incidence and prevalence statistics. DisMod-MR 2.1 is a meta-regression tool founded on the Bayesian statistical framework. As described from the official and previous literature (9, 10), its construction process is delineated as follows: First, multi-source data within the GBD database, encompassing incidence, prevalence, mortality rates and others, are integrated and preprocessed. These data are derived from global surveys, hospital records, literature reports, and monitoring systems. By means of standardization and correction of the data, the consistency of data quality is ensured. Subsequently, the DisMod-MR 2.1 model structure is established. This model is predicated on the natural history of diseases and categorizes disease states into three types: healthy, diseased, and deceased. The model depicts the transitions among these states via differential equations, including incidence, remission rate, excess mortality rate, and background mortality rate. Under the Bayesian meta-regression framework, the data are stratified by geographical regions, age, gender, etc., and covariates adjustments and random effects are incorporated to account for heterogeneity. Concurrently, rational prior distributions are set for the model parameters, and the posterior distributions are updated in combination with new data. To quantify the uncertainty, the Markov Chain Monte Carlo (MCMC) method is employed to estimate the posterior distribution, thereby generating uncertainty intervals for the parameters. Ultimately, the model outputs age-gender-region-specific disease parameter estimations and validates the rationality of the model through external data.

The diagnosis of pneumoconiosis in the Global Burden of Disease (GBD) 2021 is based on the 10th edition of the International Classification of Diseases (ICD-10). Pneumoconiosis (J60-J65.0, J92.0) encompasses silicosis (J62-J62.9), asbestosis (J61-J61.0, J92.0), coal workers’ pneumoconiosis (J60-60.0), and other forms of pneumoconiosis (J63-J65.0).

### Socio-demographic index

The SDI is a comprehensive metric introduced by the Institute for Health Metrics and Evaluation in the United States in 2015. This index assesses a nation’s level of development while highlighting the interplay between social progress and health outcomes. Essentially, it is the geometric average of total fertility rates for individuals under 25 years of age, the mean level of education for those aged 15 and older, as well as the per capita income of the lagged distribution. The value of 0 signifies the lowest levels of income and years of education, as well as the highest fertility rate. Conversely, the value of 1 indicates the highest levels of income and years of education, accompanied by the lowest fertility rate (11). As a consequence of the SDI values, 204 countries and territories have been categorized into five distinct quintiles: high SDI, high-middle SDI, middle SDI, low-middle SDI, and low SDI.

### Stratified analysis

Stratification was conducted based on age (15–19 years, in 5-year intervals up to 95 years and over), calendar year (1990–2021), and geographic region. According to the GBD study, the world is categorized into 21 distinct geographical regions. Age-standardized rate (ASR) has been employed to assess the burden of pneumoconiosis over time, across different regions, and among various age groups, including both age-standardized incidence (ASIR) and age-standardized mortality rates (ASMR). The purpose of age standardization is to eliminate the impact of population age structure, thereby enhancing the comparability of research indicators. Pearson correlation analysis was conducted to examine the association between ASRs for pneumoconiosis and the SDI across 204 countries and territories globally, with the objective of identifying determinants of the changing disease burden.

### Analyses of decomposition

To assess the influence of population growth, aging demographics, and evolving epidemiological trends on the changing burden of pneumoconiosis, we utilized a methodology established by Das Gupta (12). In this study, we employed mathematical methods to isolate the standardized effect of each multiplicative factor, thereby quantifying the specific contribution of each component to the variation in pneumoconiosis disease burden.

### Statistical analyses

Estimates of the pneumoconiosis burden, including 95% uncertainty intervals (UIs), are sourced from the GBD 2021 database (see text footnote 1, respectively). The incidence and death rates are presented as predictions, expressed in terms of individuals per 100,000 population, along with their respective 95% UIs. This study was conducted using R software (version 4.4.1; Bell Laboratories, Lucent Technologies). All hypotheses were tested on a two-sided basis with a significance level set at 0.05.

## Results

### The disease burden of pneumoconiosis incidence globally, in SDI quintiles and 21 GBD regions

The global incidence number of pneumoconiosis patients was 42,188 (95% UI: 35,786, 48,913) in 1990, rising to 62,866 (95% UI: 54, 617, 71,103) by the year 2021, as shown in Table 1. It is estimated that in 1990, the ASIR of pneumoconiosis patients worldwide was 1.029 (95% UI: 0.897–1.185) per 100,000 population. However, by 2021, the ASIR had decreased to 0.736 (95% UI: 0.639–0.832) per 100,000 population, reflecting a reduction of 28.5% (95% UI: −31.5, −25.3) compared to that of in 1990.

**Table 1:** The incidence cases and ASIR of pneumoconiosis in 1990 and 2021.

Location	1990		2021		Percentage change in ASRs from 1990 to 2021
	Case (95% UI)	ASRs per 100,000 (95% UI)	Case (95% UI)	ASRs per 100,000 (95% UI)	
Global	42,188 (35,786,48,913)	1.029 (0.879,1.185)	62,866 (54,617,71,103)	0.736 (0.639,0.832)	−28.5 (−31.5, −25.3)
Socio-demographic index					
High SDI	10,324 (8,804,11,892)	0.940 (0.807,1.078)	14,287 (12,639,16,091)	0.701 (0.618,0.789)	−25.4 (−29, −21.5)
High-middle SDI	12,194 (10,331,14,138)	1.204 (1.025,1.392)	16,083 (13,837,18,334)	0.839 (0.725,0.957)	−30.4 (−33.3, −27.1)
Middle SDI	14,440 (12,125,16,944)	1.281 (1.081,1.505)	22,871 (19,521,26,182)	0.849 (0.730,0.971)	−33.7 (−37, −30.2)
Low-middle SDI	3,995 (3,344,4,699)	0.598 (0.505,0.694)	7,285 (6,236,8,482)	0.480 (0.415,0.548)	−19.8 (−23.4, −16.1)
Low SDI	1,201 (994,1,408)	0.503 (0.426,0.583)	2,311 (1977,2,682)	0.408 (0.354,0.463)	−18.8 (−22.7, −14.8)
GBD regions					
High-income Asia Pacific	1809 (1,537,2088)	0.901 (0.769,1.033)	2,873 (2,540,3,255)	0.620 (0.548,0.702)	−31.2 (−35.5, −26.4)
High-income North America	2,699 (2,187,3,207)	0.769 (0.623,0.914)	4,505 (3,866,5,189)	0.690 (0.593,0.786)	−10.2 (−17.4, −2.8)
Western Europe	5,887 (5,125,6,672)	1.021 (0.892,1.149)	5,261 (4,762,5,840)	0.564 (0.505,0.631)	−44.7 (−47.8, −40.8)
Australasia	85 (76,95)	0.359 (0.322,0.401)	295 (271,325)	0.518 (0.474,0.572)	44.3 (33.4,55.7)
Andean Latin America	128 (111,146)	0.621 (0.539,0.703)	252 (222,284)	0.425 (0.376,0.478)	−31.5 (−35.8, −27.4)
Tropical Latin America	656 (547,785)	0.625 (0.522,0.736)	1,263 (1,102,1,435)	0.494 (0.431,0.562)	−20.8 (−26.8, −14.6)
Central Latin America	901 (747,1,077)	0.938 (0.782,1.102)	1,698 (1,414,2004)	0.661 (0.554,0.776)	−29.5 (−33.1, −26.1)
Southern Latin America	304 (271,339)	0.652 (0.582,0.725)	418 (377,466)	0.484 (0.434,0.540)	−25.8 (−29.7, −21.5)
Caribbean	62 (49,77)	0.230 (0.182,0.281)	94 (73,119)	0.178 (0.138,0.225)	−22.5 (−28.8, −16.0)
Central Europe	1,392 (1,201,1,599)	0.927 (0.804,1.056)	965 (833,1,121)	0.506 (0.434,0.588)	−45.4 (−49.2, −40.9)
Eastern Europe	1,216 (973,1,462)	0.444 (0.357,0.532)	920 (753,1,088)	0.290 (0.238,0.344)	−34.7 (−39.1, −29.8)
Central Asia	208 (173,251)	0.425 (0.355,0.501)	324 (273,384)	0.389 (0.332,0.447)	−8.5 (−13.9, −2.8)
North Africa and Middle East	797 (639,999)	0.358 (0.293,0.438)	1,666 (1,337,2052)	0.316 (0.262,0.381)	−11.5 (−16.1, −6.5)
South Asia	4,071 (3,384,4,775)	0.660 (0.557,0.773)	7,783 (6,645,9,083)	0.500 (0.433,0.577)	−24.2 (−28.8, −19.0)
Southeast Asia	1,042 (815,1,325)	0.319 (0.251,0.401)	2,173 (1721,2,700)	0.298 (0.239,0.367)	−6.7 (−12.4, −0.8)
East Asia	20,006 (16,643,23,630)	2.183 (1.820,2.556)	30,658 (26,242,35,283)	1.413 (1.215,1.624)	−35.3 (−38.6, −32.0)
Oceania	16 (12,20)	0.526 (0.431,0.626)	43 (36,51)	0.642 (0.543,0.747)	22.2 (6.5,44.2)
Western Sub-Saharan Africa	155 (116,199)	0.148 (0.113,0.189)	331 (246,433)	0.125 (0.095,0.158)	−15.4 (−21.7, −9.1)
Eastern Sub-Saharan Africa	414 (342,482)	0.550 (0.456,0.633)	727 (623,835)	0.408 (0.355,0.459)	−25.7 (−29.1, −21.8)
Central Sub-Saharan Africa	114 (94,132)	0.541 (0.459,0.619)	233 (198,271)	0.432 (0.378,0.488)	−20.2 (−24.8, −15.7)
Southern Sub-Saharan Africa	226 (189,265)	0.814 (0.682,0.952)	384 (332,438)	0.691 (0.601,0.783)	−15.1 (−19.4, −10.3)

**Figure 1:**
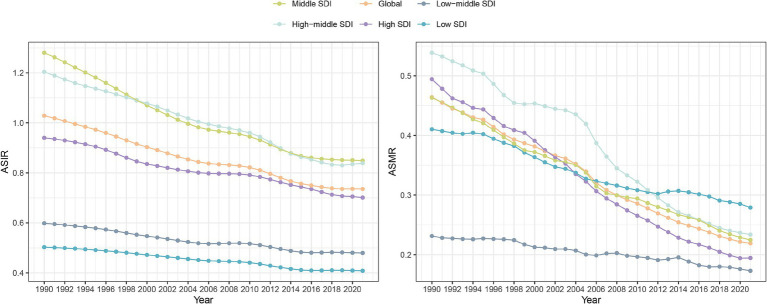
Changes in ASIR and ASMR of pneumoconiosis in SDI quintiles and globally from 1990 to 2021.

In the SDI quintiles, the middle SDI quintile experienced the most substantial decline of 33.7% from 1990 to 2021 (95% UI: −37.0, −30.2). A smaller reduction was observed in the low-middle and low SDI quintiles, with decreases of 19.8% (95% UI: −23.4, −16.1) and 18.8% (95% UI: −22.7, −14.8), respectively (Table 1). The high SDI and high-middle SDI quintiles experienced ASIR decreases of 25.4% (95% UI: −29, −21.5) and 30.4% (95% UI: −33.3, −27.1), respectively. Figure 1 illustrates the ASIR for each year from 1990 to 2021 including both global and SDI quintiles, with the legend sorted in accordance with the ASIR value for the year 2021. As illustrated, both the global and SDI quintile ASIRs have shown a decline from 1990 to 2021. However the ASIRs for the middle SDI and high-middle SDI quintiles remain higher than that of the global average. In contrast, the ASIRs for the low-middle SDI and low SDI quintile are comparatively lower.

**Figure 2:**
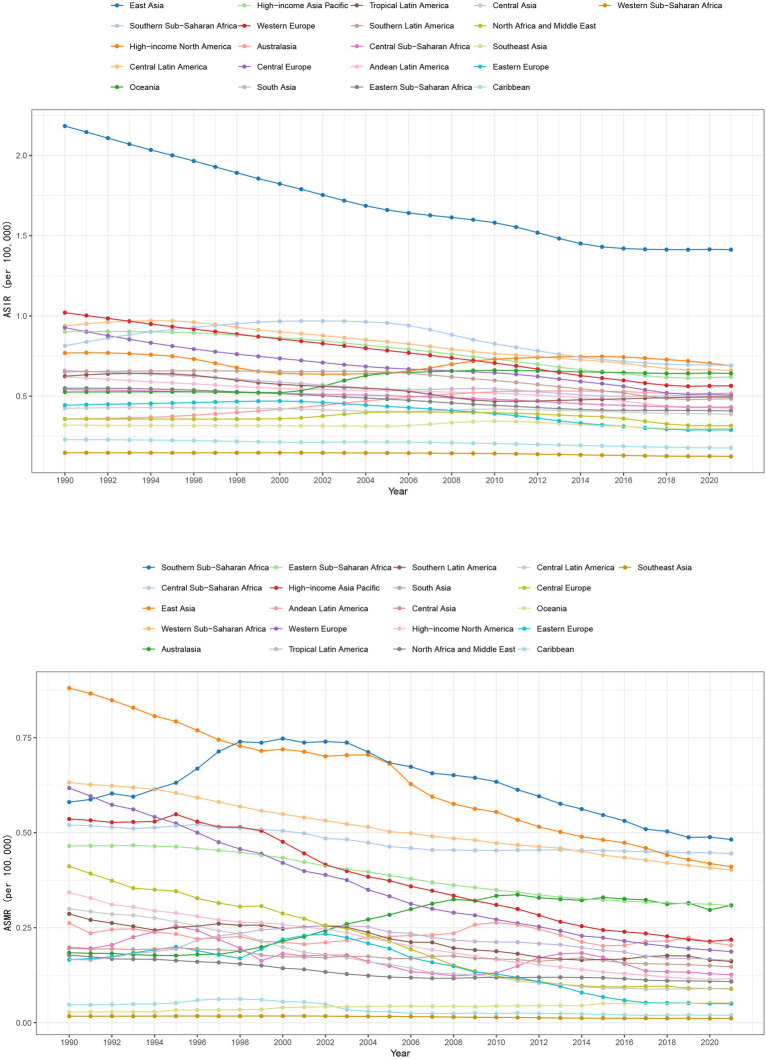
Changes in ASIR and ASMR of pneumoconiosis in GBD regions from 1990 to 2021.

Over the period 1990–2021, ASIR declined in most of the GBD regions. In Australasia and Oceania, however, a significant increase was observed, with ASIR increasing by 44.3% (95% UI: 33.4, 55.7) and 22.2% (95% UI: 6.5, 44.2), respectively (Table 1 and Figure 2). From 1990 to 2021, the ASIR of East Asia, which had the highest ASIR in 1990, decreased by 35.3% (95% UI: −38.6, −32.0). Meanwhile, in Central Europe, the ASIR declined by 45.4% (95% UI: −49.2, −40.9). Western Europe followed closely with a reduction of 44.7% (85% UI: −47.8, −40.8). The ASIR for each GBD region from 1990 to 2021 is illustrated in Figure 2. Legends are sorted according to ASIR estimations for 2021.

### The disease burden of pneumoconiosis deaths globally, in SDI quintile and 21 GBD regions

The number of death attributed to pneumoconiosis globally rose from 17,471 (95% UI: 15,589, 19,436) in 1990 to 18,323 (95% UI: 16,041, 20,916) in 2021. Following age standardization, the global ASMR decreased from 0.464 (95% UI: 0.416, 0.515) per 100,000 population in 1990 to 0.219 (95% UI: 0.192, 0.249) per 100,000 population in 2021. This represents a reduction of 52.8% (95% UI: −59.9, −44.4) over this period.

In the SDI quintiles, the most significant reduction in ASMR was observed in the high SDI quintile, with a decrease of 60.7% (95% UI: −63.7, −57.6). This was followed by a reduction of 56.6% (95% UI: −66.2, −43.9) in the high-middle SDI quintile, a decline of 51.5% (95% UI: −64.1, −35.4) in the middle SDI quintile, and a decrease of 25.2% (95% UI: −38.4, −5.0) in the low SDI quintile. As illustrated in Figure 1, the ASMR is presented for each year from 1990 to 2021, both globally and categorized by SDI quintiles. The ASMR for global and SDI quintiles exhibited a decline from 1990 to 2021. Notably, the baseline ASMR was highest in the high-middle SDI quintile; however, it ultimately fell below the 2021 level observed in the middle SDI quintile. Nonetheless, ASMR remained consistently low within the low-middle SDI and low SDI quintiles throughout this period.

The ASMR exhibited a decline in most GBD regions from 1990 to 2021. However, increases were observed in Australasia and Oceania, with rises of 67.8% (95% UI: 41.2, 105.3) and 86.1% (95% UI: 0.9, 259.9), respectively. Central Europe experienced the most significant reduction in ASMR, with a decrease of 78.4% (95% UI: −81.8, −74.8). This was followed by Eastern Europe, which saw a decline of 69.9% (95% UI: −75.1, −61.7), Central Latin America at a reduction of 69.8% (95% UI: −73.3, −66.2), and Western Europe with a decrease of 69.7% (95% UI: −72.5, −66.6). Figure 2 presents the ASMR for the 21 GBD regions from 1990 to 2021, with the legend sorted according to the ASMR values for the year 2021. Although East Asia continues to exhibit the highest in 1990, this downward trend is clearly observable. In 2021, the ASMR is already lower than that of both Southern Sub-Saharan Africa and Central Sub-Saharan Africa. In comparison to 1990, the ASMR in Australasia exhibited a significant increase in the year 2021 (Table 2).

**Table 2:** The deaths cases and ASMR of pneumoconiosis in 1990 and 2021.

Location	1990		2021		Percentage change in ASRs from 1990 to 2021
	Case (95% UI)	ASRs per 100,000 (95% UI)	Case (95% UI)	ASRs per 100,000 (95% UI)	
Global	17,471 (15,589,19,436)	0.464 (0.416,0.515)	18,323 (16,041,20,916)	0.219 (0.192,0.249)	−52.8 (−59.9, −44.4)
Socio-demographic index					
High SDI	5,654 (5,348,5,941)	0.494 (0.468,0.519)	4,643 (4,157,4,977)	0.194 (0.176,0.208)	−60.7 (−63.7, −57.6)
High-middle SDI	5,135 (4,545,5,781)	0.539 (0.479,0.606)	4,537 (3,700,5,540)	0.234 (0.191,0.285)	−56.6 (−66.2, −43.9)
Middle SDI	4,647 (3,838,5,606)	0.463 (0.387,0.553)	5,701 (4,553,7,068)	0.225 (0.181,0.277)	−51.5 (−64.1, −35.4)
Low-middle SDI	1,245 (794,1706)	0.231 (0.148,0.320)	2,250 (1,666,2,862)	0.173 (0.127,0.222)	−25.2 (−38.4, −5.0)
Low SDI	777 (450,1,255)	0.410 (0.238,0.661)	1,185 (636,1,951)	0.279 (0.148,0.462)	−32 (−44.6, −10.5)
GBD regions					
High-income Asia Pacific	1,072 (998,1,148)	0.536 (0.499,0.574)	1,270 (1,105,1,413)	0.219 (0.192,0.245)	−59.2 (−64.1, −53.9)
High-income North America	1,296 (1,194,1,385)	0.343 (0.316,0.365)	830 (739,899)	0.116 (0.103,0.125)	−66.2 (−69.0, −63.4)
Western Europe	3,808 (3,563,4,044)	0.618 (0.580,0.655)	2,177 (1938,2,386)	0.187 (0.169,0.204)	−69.7 (−72.5, −66.8)
Australasia	45 (39,51)	0.184 (0.161,0.21)	190 (163,219)	0.309 (0.266,0.356)	67.8 (41.2, 105.3)
Andean Latin America	52 (38,67)	0.262 (0.196,0.335)	117 (85,162)	0.204 (0.148,0.283)	−22.4 (−49.2, 10.6)
Tropical Latin America	154 (143,166)	0.167 (0.153,0.182)	414 (380,446)	0.164 (0.15,0.177)	−1.8 (−11.7, 9.9)
Central Latin America	227 (215,239)	0.300 (0.283,0.316)	218 (190,244)	0.091 (0.079,0.102)	−69.8 (−73.3, −66.2)
Southern Latin America	129 (114,149)	0.287 (0.253,0.329)	146 (126,168)	0.161 (0.138,0.185)	−43.7 (−53.5, −32.4)
Caribbean	11 (10,13)	0.047 (0.041,0.054)	11 (9,13)	0.02 (0.016,0.025)	−58.2 (−66.6, −48.8)
Central Europe	616 (558,681)	0.412 (0.374,0.455)	207 (185,234)	0.089 (0.079,0.100)	−78.4 (−81.8, −74.8)
Eastern Europe	459 (377,540)	0.166 (0.137,0.194)	178 (160,196)	0.050 (0.045,0.055)	−69.9 (−75.1, −61.7)
Central Asia	90 (68,119)	0.198 (0.149,0.263)	94 (69,129)	0.127 (0.093,0.172)	−36.1 (−59.9, −0.4)
North Africa and Middle East	285 (217,404)	0.178 (0.133,0.265)	452 (350,594)	0.108 (0.085,0.142)	−39.1 (−53.5, −18.0)
South Asia	947 (517,1,446)	0.196 (0.108,0.298)	1919 (1,301,2,660)	0.147 (0.101,0.204)	−24.9 (−43.4, 10.1)
Southeast Asia	38 (26,53)	0.017 (0.012,0.024)	65 (43,93)	0.011 (0.008,0.016)	−34.3 (−53.9, 1.4)
East Asia	7,233 (5,897,8,846)	0.881 (0.723,1.063)	8,496 (6,606,10,957)	0.411 (0.321,0.524)	−53.4 (−67.1, −35.8)
Oceania	0 (0,1)	0.028 (0.011,0.056)	3 (1,6)	0.053 (0.017,0.119)	86.1 (0.9, 259.9)
Western Sub-Saharan Africa	477 (294,757)	0.632 (0.386,0.996)	644 (362,1,014)	0.402 (0.228,0.623)	−36.4 (−49.3,-19.8)
Eastern Sub-Saharan Africa	297 (157,510)	0.465 (0.241,0.788)	441 (207,748)	0.308 (0.141,0.524)	−33.8 (−47.6, −13.6)
Central Sub-Saharan Africa	95 (46,163)	0.520 (0.240,0.927)	199 (83,398)	0.445 (0.175,1.008)	−14.4 (−44.4, 33.6)
Southern Sub-Saharan Africa	142 (107,179)	0.581 (0.435,0.746)	251 (216,287)	0.482 (0.418,0.561)	−17.0 (−35.6, 5.6)

### The age distribution of ASIR and ASMR of pneumoconiosis in the world and the GBD regions

**Figure 3:**
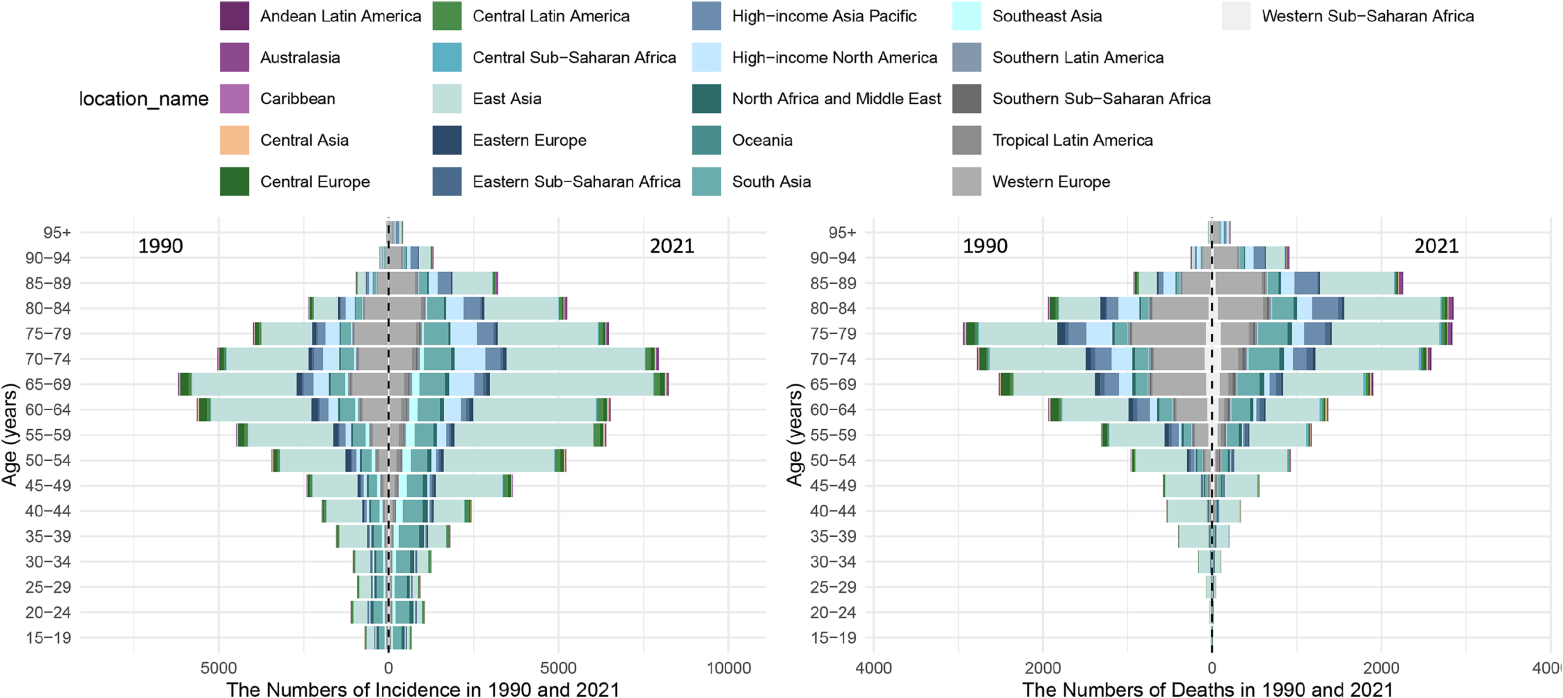
Age Distribution of Pneumoconiosis Incidence and deaths Across 21 GBD Regions and Globally in 1990 and 2021.

As demonstrated in Figure 3 and Supplementary Table 1, the incidence of pneumoconiosis numbers worldwide has shown a consistent increase with age from 1990 to 2021, peaking within the 65–69 age group before experiencing a gradual decline. Notably, after reaching the age range of 25–29, all subsequent age groups showed a higher number of cases in 2021 than in 1990. There was also an increase with advancing age in the number of deaths attributed to pneumoconiosis worldwide in 1990 and 2021, with peaks observed in the age groups of 75–79 and 80–84, respectively. Before the 75–79 age group, the number of deaths estimated in 2021 was lower than that in 1990. Conversely, for those aged beyond this group, the mortality numbers were higher compared to those estimated in 1990. A comparable age distribution is observed in East Asia, where the burden of disease is most pronounced. In Western Europe, where the burden of disease is also significant, patients with pneumoconiosis reach their peak incidence and mortality in the ages of 80–84 group. In comparison to global trends, the peak ages for both the incidence and mortality of pneumoconiosis have shifted to later years.

### The relationship between SDI and pneumoconiosis disease burden

**Figure 4:**
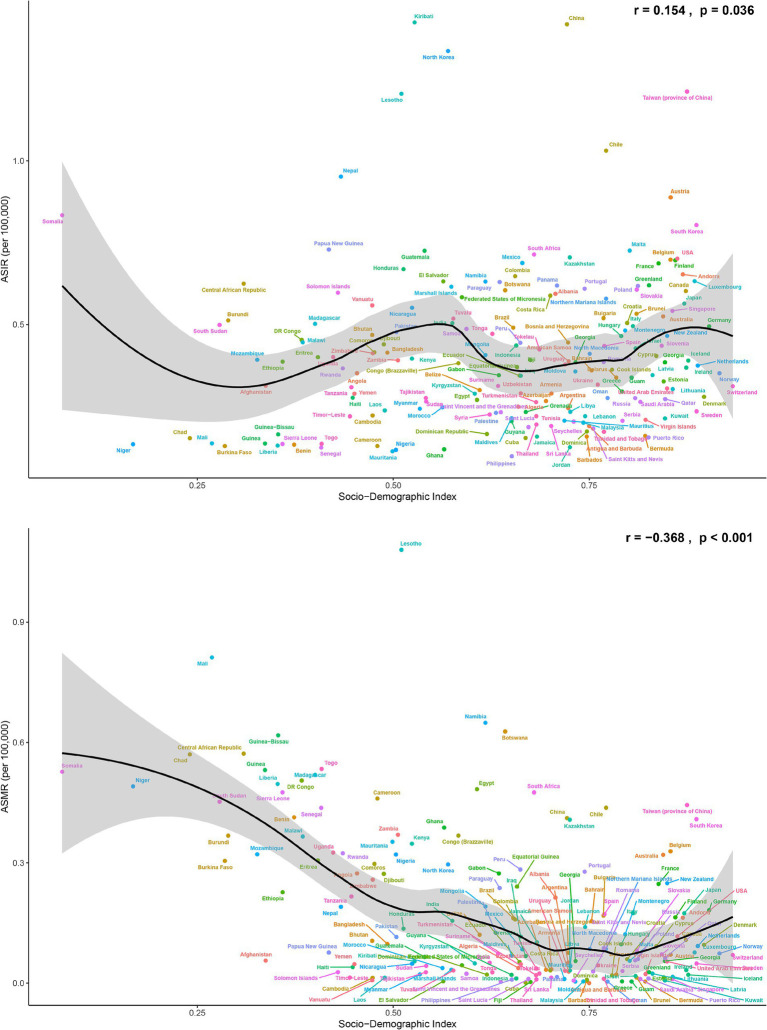
The ASIR and ASMR of pneumoconiosis in 204 countries and territories in 2021, with the corresponding values of SDI.

Data from 2021 indicate that among 204 countries and territories, the ASIR of pneumoconiosis increased with rising SDI (*r* = 0.154, *p* = 0.036), as shown in Figure 4. The ASIR was notably high in Kiribati (1.42 per 100,000 population), China (1.42 per 100,000 population), North Korea (1.34 per 100,000 population), Lesotho (1.2 per 100,000 population), and Chile (1.03 per 100,000 population). Somalia (0.83 per 100,000 population) had the highest incidence rate in low SDI quintiles, while Austria (0.89 per 100,000 population), South Korea (0.80 per 100,000 population), the United States (0.7 per 100,000 population), and France (0.69 per 100,000 population) exhibited relatively high rates in high SDI quintiles. The ASMR of pneumoconiosis showed a negative correlation with SDI (*r* = −0.368, *p* < 0.001). Lesotho recorded the highest mortality rate at 1.08 per 100,000 population, followed by Mali and Namibia. Notably, mortality rates in high SDI regions demonstrated an upward trend compared to high-middle SDI quintiles.

### Analyses of decomposition

**Figure 5:**
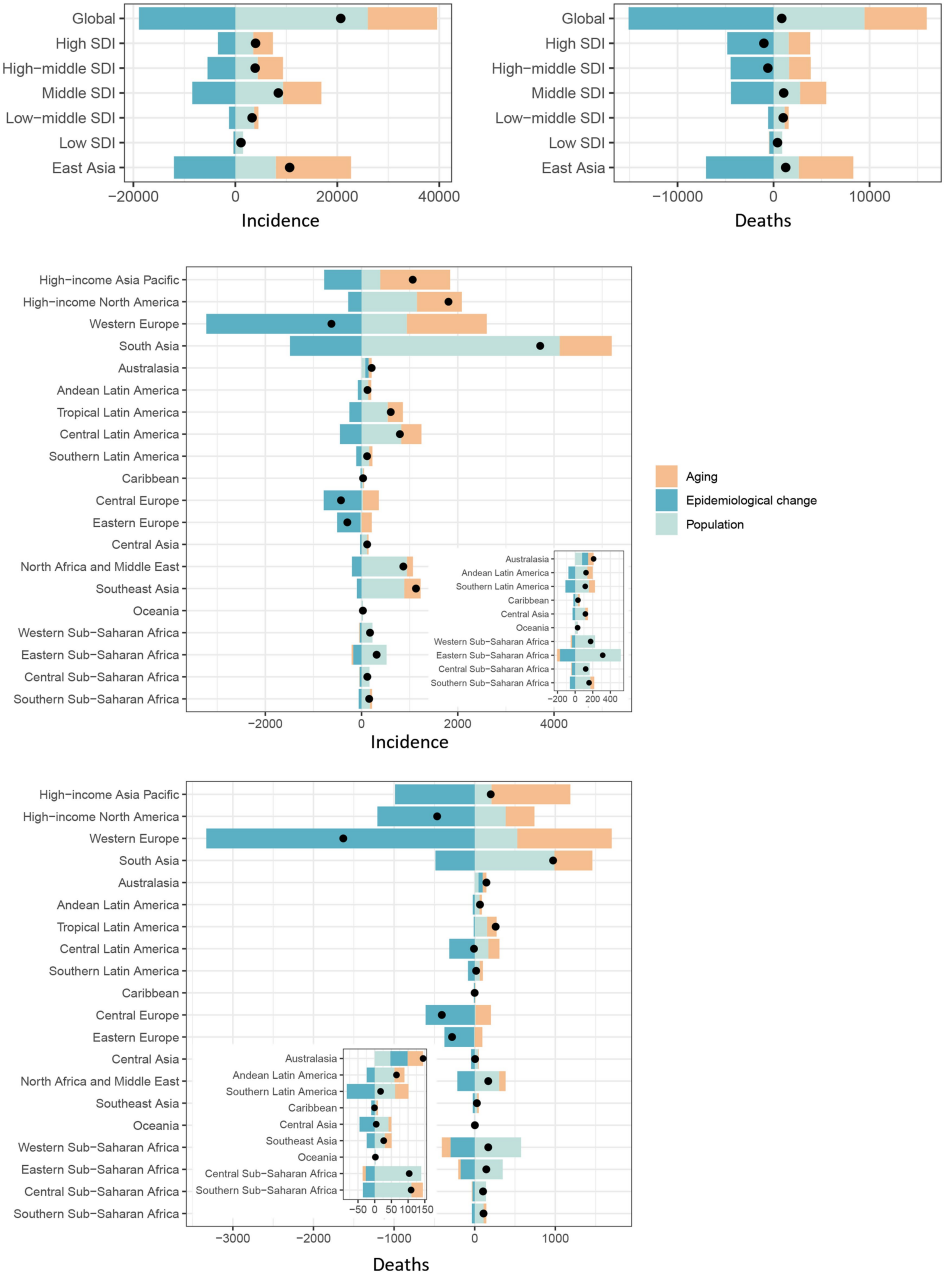
Changes in pneumoconiosis incidence and deaths in according to population-level determinants: impact of population growth, aging, and epidemiological dynamics from 1990 to 2021.

In general, pneumoconiosis incidence and mortality have increased over the past 32 years, with the largest increases observed in the middle SDI quintile (Supplementary Tables 2, 3; Figure 5). In East Asia, the decomposition analysis chart has been relocated to the SDI quintile regions due to a higher population than other GBD regions (Figure 5). Following East Asia, South Asia experienced the greatest increase in both incidences and deaths. Both the number of incidences and deaths in Western Europe decreased the most. Supplementary Tables 2, 3 provide detailed values. In general, aging and population growth are the principal factors contributing to pneumoconiosis cases rise. Epidemiological changes have negatively impacted the increasing number of this disease. The rise in pneumoconiosis cases in Australasia and Oceania may be positively influenced by epidemiological changes.

The black dot signifies the cumulative change resulting from the three components. For each constituent, a positive value denotes an increment in pneumoconiosis incidence/deaths associated with that factor, with the extent of the value reflecting the magnitude of the increase. Conversely, a negative value signifies a reduction in pneumoconiosis incidence/deaths linked to the respective factor, with the value’s size indicating the degree of the decrease.

## Discussion

During the period from 1990 to 2021, the global and regional disease burden of pneumoconiosis has changed significantly. The primary contributor to occupational mortality and disability is occupational pneumoconiosis. This study utilizes GBD 2021 data to estimate the incidence and deaths in the world from 1990 to 2021, while several studies have previously estimated the prevalence and incidence of pneumoconiosis and its subspecies silicosis (3), asbestosis (13), and coal workers’ pneumoconiosis (14) in specific regions using previous data.

This study revealed that the incidence of pneumoconiosis rose from 42,188 cases in 1990 to 62,866 cases in 2021. Concurrently, the number of deaths from this disease increased from 17,471 in 1990 to 18,323 in 2021. However, both the ASIR and ASMR for pneumoconiosis have shown a continuous decline over this period. As illustrated in Figure 5, the rise in both the incidence and death numbers of pneumoconiosis on a global and regional scale can be primarily attributed to the increase in the global population, as well as demographic aging. The age-standardized burden of pneumoconiosis has diminished over the past 32 years, signifying advancements in the prevention and treatment of this disease on a global scale (15). Other data further support this conclusion. For instance, according to reports from Ontario, Canada, the incidence of silicosis fell from 0.42 cases per 100,000 people in 1996–2000 to 0.06 cases per 100,000 population in 2016–2019. A similar trend was observed for asbestosis, decreasing from 1.66 cases per 100,000 population to 0.51 cases per 100,000 population (16). The Italian health authorities collected discharge data from nearly all hospitals nationwide. From 2001 to 2015, hospital admissions due to asbestosis or silicosis declined significantly by 68.1%, with non-urgent hospitalizations decreasing by 83.6%. Moreover, the reduction in in-hospital mortality was even more pronounced (17). The US CDC analyzed domestic pneumoconiosis mortality data from 1999 to 2018. During this period, overall pneumoconiosis deaths number decreased by 40.4%, with coal workers’ pneumoconiosis deaths declining by 69.6% and silicosis deaths dropping by 53.0% (18).

In 2021, the AISR and ASMR of pneumoconiosis in the middle SDI and high-middle SDI quintiles were higher than the global average, whereas those in the low-middle SDI and low SDI quintiles were lower than the global average (Figure 1). In accordance with previous studies, countries with middle to high SDI levels experience a disproportionately high burden of pneumoconiosis (2, 3). This observation may be explained by rapid urbanization and industrialization in middle SDI and high-middle SDI quintiles, accelerating occupational dust exposure and raising disease incidence. As well, improved medical resources and public awareness may enhance the detection of pneumoconiosis. The use of high-voltage chest radiography, computed tomography (CT), and multidisciplinary team evaluations play a critical role in diagnosing pneumoconiosis (19, 20). In contrast, low SDI quintiles may be underdiagnosed of pneumoconiosis due to limited access to modern diagnostic technologies and underdeveloped healthcare systems.

In most of the GBD regions, the ASIR and ASMR decreased from 1990 to 2021. The Australasia and Oceania regions, however, both rates increased, with ASIR increasing by 44.3% (95 UI: 33.4, 55.7) and 22.2% (95 UI: 6.5, 44.2), and ASMR increasing by 67.8% (95 UI: 41.2, 105.3) and 86.1% (9 UI: 0.9, 259.9), respectively.

Figure 4 presents the correlation analysis of ASIR and ASMR with SDI values across 204 countries and territories globally. The ASIR exhibits a weak positive correlation with SDI, while the ASMR shows a negative correlation. Pneumoconiosis is highly prevalent in industrial settings, particularly in industries with high levels of industrialization such as mining, construction, and manufacturing. Industrial development levels vary significantly among countries worldwide. Nations like the United States, France, Austria, and South Korea have undergone multiple industrial revolutions and completed the industrialization process. China and Chile are currently at an intermediate stage of industrialization, while countries such as Lesotho, Kiribati, North Korea, and Somalia remain at a low level of industrialization due to factors such as isolation, poverty, and geographical location. Countries with high or post-industrialization levels generally exhibit relatively lower incidences of pneumoconiosis, likely due to stringent occupational health protection measures and advanced medical technologies. However, some cases still occur. Countries with low levels of industrialization tend to have lower pneumoconiosis incidences, possibly due to weaker industrial foundations. Nevertheless, certain low-industrialization countries experience relatively higher incidence, potentially due to specific industrial activities or inadequate protective measures. Intermediate-stage industrialized countries show the highest incidence of pneumoconiosis, likely because of frequent industrial activities, especially in high-risk sectors such as mining, construction, and manufacturing. The higher the SDI values, the lower the ASMR of pneumoconiosis, indicating that favorable medical conditions and strict occupational health policies can effectively reduce mortality. However, in high SDI quintiles, the mortality rate has shown a relative increase compared to high-middle SDI quintiles, which may be attributed to factors such as aging population, increased burden of chronic diseases, and historical industrial issues.

Based on the decomposition analysis of Figure 5, changes in the epidemiology of pneumoconiosis in most regions have been negative, resulting in a decrease in incidence and death numbers. In Australasia, pneumoconiosis epidemiological changes are positive, leading to increased incidence and death numbers. Silicosis cases in Australia increased significantly in 2019, which was closely related to the rapid expansion of the artificial stone industry. Since 2015, approximately 580 workers engaged in the processing of artificial stones have been diagnosed with pneumoconiosis in Australia (21). Monash University reports that up to 25% of workers in Victoria’s artificial stone countertop industry suffer from pneumoconiosis. Artificial stone is banned in Australia for the first time in response to the surge in silicosis cases (22). The nationwide ban came into effect in most states and territories on July 1, 2024. The ban on the use and import of engineered stone products in Australia is aimed to diminish the risk of workers developing silicosis and other silica dust-related diseases. By taking these measures, the Australian government shows that it takes workers’ health seriously and has taken appropriate action to safeguard it.

The alarming increase in pneumoconiosis cases has not been confined to Australia alone in recent decades. In China, the rapid expansion of the mining industry has exposed a significant number of workers to occupational hazards. During the past 32 years, the ASIR of pneumoconiosis has consistently been higher in East Asia than in other regions, and it remained elevated in 2021. As the SDI increased, both ASIR and ASMR in East Asia decreased markedly due to economic development, policy implementation, improved medical care, and increased public health awareness (23), as illustrated in Figure 4. The disease burden of pneumoconiosis cannot, however, be ignored at this time, given the long latency period of pneumoconiosis and the ongoing challenges in comprehensive regulation and prevention (24).

As shown in Figure 2, the regions with significant declines in ASIR include Western Europe (−44.7, 95% UI: −47.8% to −40.8%) and Central Europe (−45.4, 95% UI: −49.2% to −40.9%). The region with the largest reduction in ASMR is Central Europe (−78.4, 95% UI: −81.8% to −74.8%), followed by Eastern Europe (−69.9, 95% UI: −75.1% to −61.7%), Central Latin America (−69.8, 95% UI: −73.3% to −66.2%), and Western Europe (−69.7, 95% UI: −72.5% to −66.8%). According to the decomposition analysis presented in Figure 5, the reductions in ASIR and ASMR in Western Europe were primarily attributed to epidemiological changes, which accounted for a substantial proportion of the decline. Overall, European regions are at the forefront in terms of pneumoconiosis prevention and treatment. In Europe, such as Italy and Spain, the primary causes of pneumoconiosis are the inhalation of asbestos fibers and silica dust. In Italy, the National Institute for Insurance against Accidents at Work coordinates pneumoconiosis prevention and control efforts based on the Occupational Health and Safety Act. A national silicosis surveillance network has been established to monitor cases. In 1992, Italy enacted an asbestos ban and initiated a comprehensive removal program, subsequently promoting the use of substitutes and preventive measures. In Spain, the focus is on controlling dust sources and reducing airborne transmission. Multiple regulations have been implemented to restrict dust-generating industries and minimize worker exposure, thereby reducing the risk of pneumoconiosis.

The global burden of pneumoconiosis increased with age from 1990 to 2021. The incidence number peaked in the 65–69 age group, and in each age group above 25–29 years, the number of incidence in 2021 was higher than in 1990. It can be attributed to the global aging population and overall population growth over the past 32 years. Silicosis, the most prevalent and severe form of pneumoconiosis, manifests clinically several years after silica dust exposure, with symptoms such as cough, expectoration, and dyspnea. Even after cessation of exposure, silicosis continues to cause progressive lung damage. Consequently, the age of disease onset is significantly delayed compared to the age of initial dust exposure. Figure 3 shows that the number of deaths from pneumoconiosis in the world increased progressively with age in 1990 and 2021, peaking in the 75–79 age group in 1990 and the 80–84 age group in 2021. In the East Asian region, which accounts for the largest population share, the age distribution of pneumoconiosis incidence and deaths is in line with the global pattern. Compared with the global peak, pneumoconiosis incidence and death in Western Europe, the second-largest affected region has been notably delayed since 2021. It can be attributed to the cumulative clinical experience of occupational health professionals over the past 32 years, as well as advancements in diagnostic and therapeutic technologies. This advance is even more evident In Western Europe. Additionally, this trend underscores the challenges of implementing relevant social security measures in an aging population.

At present, treatment options for pneumoconiosis remain limited. Comprehensive management primarily focuses on common clinical symptoms of patients, including cough, chest pain, and dyspnea, which may provide relief in symptoms and improve quality of life. Most workers do not wear protective equipment during work, and new hazardous materials often go undetected in a timely manner. These factors may result in harmful dust exposure. The prevention, early diagnosis and advanced treatment strategies of pneumoconiosis are essential to addressing the problem. In order to improve the quality of life and prolong survival in patients, researchers need to identify therapeutic targets and develop innovative therapeutic strategies. In the near future, some potential treatments, such as antifibrotic drugs (25, 26), may be clinically applicable. With the advent of high-throughput omics technologies, rapid advancements in bioinformatics, and gene editing technology, in-depth research on pneumoconiosis is now possible (27).

Pneumoconiosis is an incurable condition, underscoring the critical importance of prevention. The disease can lead to a decline in lung function even before chest imaging reveals any abnormalities. For monitoring everyone to 3 years, some scholars have proposed using lung function tests and low-dose conventional and high-resolution computed tomography scans (28). Importantly, pulmonary fibrosis may continue to progress even after the dust exposure has ceased. A comprehensive, coordinated, and regular screening approach is therefore important for secondary and tertiary prevention strategies that can result in an improvement in quality of life and lifespan (29). While financial constraints in low- and middle-income countries may limit such efforts, this approach remains particularly vital because advanced pneumoconiosis can lead to complications such as pulmonary arterial hypertension and right heart failure (30), posing significant threats to life (31). Additionally, individuals with pneumoconiosis have a higher risk of developing concurrent conditions like tuberculosis and malignant tumors compared to the general population.

For this study, we used data from the GBD 2021 database, which is comprised of population dynamics registrations, death surveillance data, inpatient hospital registrations, insurance claims files and systematic reviews of different countries. Consequently, the data are detailed, homogeneous and highly comparable, which facilitates comparisons between regions. However, the database also has several limitations. First, all data used in this study were derived from GBD 2021, which aggregates information from diverse sources. In some low-income countries, the actual number of cases is likely to be underestimated because of a lack of reliable epidemiological data, underdiagnosis, and underreporting. Global collaboration must be strengthened, and a unified online reporting system developed for better management and data integration (32). In addition, the GBD data update process has a time lag that prevents it from quickly reflecting current health conditions and disease trends. This delay can hinder timely decision-making during public health events or outbreaks. In data-scarce situations, reliance on estimation models may introduce biases. To combat these issues, GBD conducts annual collaborative data searches with domestic partners, followed by thorough data cleaning and validation. Regular updates to GBD estimates help reduce discrepancies and lag in data integration (33).

## Conclusion

Overall, pneumoconiosis remains an important occupational health problem in the world, with a persistent and substantial disease burden. Compared to global averages, middle SDI and high-middle SDI quintiles have had higher AISR and ASMR of pneumoconiosis over the past 32 years. While East Asia has a notably high ASIR of pneumoconiosis, both the ASIR and ASMR have shown marked declines. Similarly, Europe has witnessed significant reductions in both ASIR and ASMR. This downward trend may be attributed to the emphasis on the prevention, diagnosis, and treatment of pneumoconiosis in most parts of the world. In 2021, the pneumoconiosis burden was highest among 65-69-year-olds worldwide, highlighting the latency period associated with occupational pneumoconiosis. A positive correlation was found between the ASIR of pneumoconiosis in 2021 and SDI, while a negative correlation was found between SDI and ASMR. The problem of pneumoconiosis is not only a global health challenge but also necessitates adjustments in occupational environment regulation and public health services. Pneumoconiosis burden reduction is of critical importance. There is a need for the government to establish and refine occupational health management regulations and strengthen supervision. Enterprise managers should prioritize the construction of protective facilities in the workplace, conduct regular occupational health examinations for employees, and implement effective preventive measures. The development of new technologies, the transition to dust-free operations, and increased health awareness will result in a sustained reduction in the pneumoconiosis disease burden.

## Data Availability

Publicly available datasets were analyzed in this study. This data can be found at: https://ghdx.healthdata.org/gbd-2021/.

## Footnotes

^1^ https://ghdx.healthdata.org/gbd-2021/
