# Supplementary material for: Global and regional burden of pneumoconiosis, 1990–2021: an analysis of data from the global burden of disease study 2021
Source: Front Med (Lausanne). 2025 Mar 28;12:1559540. doi: 10.3389/fmed.2025.1559540 (PMC11985417; doi:10.3389/fmed.2025.1559540)
Supplement: Supplementary file 1 [file Table_1.docx]

Supplementary Table 1 Global Age Distribution of Pneumoconiosis Incidence and Mortality in 1990 and 2021

| Age (years) | Incidence | | | | Deaths | | | |
| --- | --- | --- | --- | --- | --- | --- | --- | --- |
|  | 1990 | | 2021 | | 1990 | | 2021 | |
|  | number | Rate | number | Rate | number | Rate | number | Rate |
| 15-19 | 709.056 | 0.137 | 682.278 | 0.109 | 26.354 | 0.005 | 12.318 | 0.002 |
| 20-24 | 1,120.038 | 0.228 | 1,070.378 | 0.179 | 35.974 | 0.007 | 22.066 | 0.004 |
| 25-29 | 926.507 | 0.209 | 936.551 | 0.159 | 75.158 | 0.017 | 43.734 | 0.007 |
| 30-34 | 1,056.031 | 0.274 | 1,259.186 | 0.208 | 164.037 | 0.043 | 103.699 | 0.017 |
| 35-39 | 1,549.584 | 0.440 | 1,824.765 | 0.325 | 401.132 | 0.114 | 204.446 | 0.036 |
| 40-44 | 1,966.068 | 0.686 | 2,437.652 | 0.487 | 542.412 | 0.189 | 337.467 | 0.067 |
| 45-49 | 2,410.758 | 1.038 | 3,646.283 | 0.770 | 582.005 | 0.251 | 557.262 | 0.118 |
| 50-54 | 3,444.293 | 1.620 | 5,218.791 | 1.173 | 963.996 | 0.453 | 928.031 | 0.209 |
| 55-59 | 4,489.392 | 2.424 | 6,393.453 | 1.616 | 1,313.794 | 0.709 | 1,175.033 | 0.297 |
| 60-64 | 5,648.713 | 3.517 | 6,532.494 | 2.041 | 1,936.900 | 1.206 | 1,368.938 | 0.428 |
| 65-69 | 6,189.652 | 5.007 | 8,235.842 | 2.986 | 2,523.886 | 2.042 | 1,904.721 | 0.691 |
| 70-74 | 5,038.882 | 5.952 | 7,942.719 | 3.859 | 2,780.144 | 3.284 | 2,590.131 | 1.258 |
| 75-79 | 3,982.210 | 6.469 | 6,482.403 | 4.915 | 2,940.604 | 4.777 | 2,838.863 | 2.153 |
| 80-84 | 2,353.602 | 6.653 | 5,249.434 | 5.994 | 1,945.517 | 5.500 | 2,852.404 | 3.257 |
| 85-89 | 970.875 | 6.425 | 3,213.132 | 7.028 | 927.617 | 6.139 | 2,255.823 | 4.934 |
| 90-94 | 268.265 | 6.260 | 1,317.423 | 7.364 | 257.559 | 6.010 | 910.161 | 5.088 |
| 95+ | 64.062 | 6.292 | 423.669 | 7.773 | 54.235 | 5.327 | 217.438 | 3.989 |

Supplementary Table 2 Changes in pneumoconiosis incidence number according to population-level determinants and causes from 1990 to 2021

| Location | Overll  difference | Change due to Population-level detemminants (% contribute to the total changes) | | | | | |
| --- | --- | --- | --- | --- | --- | --- | --- |
|  |  | Aging  effect | Population  effect | Epidemiological  change effect | Aging  (%) | Population  (%) | Epidemiology  Change (%) |
| Global | 20678.46 | 13612 | 25972.9 | -18906.4 | 65.83 | 125.6 | -91.43 |
| Socio-demographic Index | | |  |  |  |  |  |
| High SDI | 3962.77 | 3876.427 | 3495.676 | -3409.33 | 97.82 | 88.21 | -86.03 |
| High-middle SDI | 3888.35 | 4950.286 | 4397.457 | -5459.4 | 127.31 | 113.09 | -140.4 |
| Middle SDI | 8430.85 | 7509.684 | 9364.268 | -8443.1 | 89.07 | 111.07 | -100.15 |
| Low-middle SDI | 3290.03 | 846.07 | 3683.496 | -1239.53 | 25.72 | 111.96 | -37.68 |
| Low SDI | 1109.86 | -58.961 | 1521.781 | -352.965 | -5.31 | 137.11 | -31.8 |
| GBD regions | |  |  |  |  |  |  |
| High-income Asia Pacific | 1063.39 | 1453.135 | 387.66 | -777.404 | 136.65 | 36.46 | -73.11 |
| High-income North America | 1806.39 | 933.998 | 1150.051 | -277.654 | 51.71 | 63.67 | -15.37 |
| Central Europe | -427.11 | 334.849 | 24.727 | -786.684 | -78.4 | -5.79 | 184.19 |
| Central Latin America | 796.98 | 421.644 | 823.262 | -447.924 | 52.91 | 103.3 | -56.2 |
| Central Sub-Saharan Africa | 119.52 | -8.168 | 165.192 | -37.502 | -6.83 | 138.21 | -31.38 |
| Eastern Sub-Saharan Africa | 313.14 | -33.839 | 518.945 | -171.966 | -10.81 | 165.72 | -54.92 |
| Southern Sub-Saharan Africa | 157.99 | 45.392 | 171.791 | -59.194 | 28.73 | 108.74 | -37.47 |
| Southern Latin America | 114.62 | 69.482 | 155.554 | -110.416 | 60.62 | 135.71 | -96.33 |
| Andean Latin America | 123.39 | 62.679 | 137.05 | -76.341 | 50.8 | 111.07 | -61.87 |
| Tropical Latin America | 606.69 | 316.579 | 542.936 | -252.823 | 52.18 | 89.49 | -41.67 |
| East Asia | 10652.06 | 14756.34 | 7936.516 | -12040.8 | 138.53 | 74.51 | -113.04 |
| South Asia | 3712.08 | 1082.155 | 4117.34 | -1487.42 | 29.15 | 110.92 | -40.07 |
| Southeast Asia | 1131.07 | 340.985 | 886.784 | -96.701 | 30.15 | 78.4 | -8.55 |
| Western Europe | -625.9 | 1660.811 | 940.467 | -3227.18 | -265.35 | -150.26 | 515.61 |
| Western Sub-Saharan Africa | 175.73 | -15.248 | 226.302 | -35.322 | -8.68 | 128.78 | -20.1 |
| Eastern Europe | -295.43 | 211.964 | -23.178 | -484.216 | -71.75 | 7.85 | 163.9 |
| North Africa and Middle East | 868.42 | 126.576 | 941.658 | -199.813 | 14.58 | 108.43 | -23.01 |
| Australasia | 209.6 | 62.948 | 78.835 | 67.821 | 30.03 | 37.61 | 32.36 |
| Caribbean | 31.92 | 19.678 | 32.12 | -19.873 | 61.65 | 100.63 | -62.26 |
| Oceania | 27.85 | 3.211 | 22.215 | 2.429 | 11.53 | 79.77 | 8.72 |

Supplementary Table 3 Changes in pneumoconiosis deaths number according to population-level determinants and causes from 1990 to 2021

| Location | Overll  difference | Change due to Population-level detemminants (% contribute to the total changes) | | | | | |
| --- | --- | --- | --- | --- | --- | --- | --- |
|  |  | Aging  effect | Population  effect | Epidemiological  change effect | Aging  (%) | Population  (%) | Epidemiology  Change (%) |
| Global | 851.21 | 6491.942 | 9465.695 | -15106.4 | 762.67 | 1112.03 | -1774.7 |
| Socio-demographic Index | | |  |  |  |  |  |
| High SDI | -1011.43 | 2238.898 | 1575.49 | -4825.82 | -221.36 | -155.77 | 477.13 |
| High-middle SDI | -597.09 | 2266.045 | 1606.282 | -4469.42 | -379.51 | -269.02 | 748.53 |
| Middle SDI | 1054.19 | 2729.32 | 2755.986 | -4431.11 | 258.9 | 261.43 | -420.33 |
| Low-middle SDI | 1005.35 | 426.759 | 1148.223 | -569.633 | 42.45 | 114.21 | -56.66 |
| Low SDI | 407.72 | -46.824 | 892.351 | -437.803 | -11.48 | 218.86 | -107.38 |
| GBD regions | |  |  |  |  |  |  |
| High-income Asia Pacific | 198.53 | 978.975 | 207.315 | -987.761 | 493.11 | 104.43 | -497.54 |
| High-income North America | -466.02 | 357.014 | 384.227 | -1207.26 | -76.61 | -82.45 | 259.06 |
| Central Europe | -408.2 | 191.824 | 9.155 | -609.183 | -46.99 | -2.24 | 149.24 |
| Central Latin America | -9.19 | 137.403 | 169.202 | -315.796 | -1495.14 | -1841.15 | 3436.3 |
| Central Sub-Saharan Africa | 103.32 | -9.434 | 139.433 | -26.674 | -9.13 | 134.95 | -25.82 |
| Eastern Sub-Saharan Africa | 143.6 | -30.265 | 347.603 | -173.735 | -21.08 | 242.06 | -120.99 |
| Southern Sub-Saharan Africa | 109.02 | 34.401 | 109.884 | -35.268 | 31.55 | 100.79 | -32.35 |
| Southern Latin America | 17.58 | 39.854 | 61.289 | -83.567 | 226.7 | 348.63 | -475.35 |
| Andean Latin America | 65.21 | 30.337 | 58.765 | -23.891 | 46.52 | 90.12 | -36.64 |
| Tropical Latin America | 259.76 | 119.795 | 152.216 | -12.247 | 46.12 | 58.6 | -4.71 |
| East Asia | 1263.56 | 5681.403 | 2621.867 | -7039.71 | 449.63 | 207.5 | -557.13 |
| South Asia | 972.25 | 471.132 | 988.478 | -487.359 | 48.46 | 101.67 | -50.13 |
| Southeast Asia | 26.95 | 20.518 | 30.172 | -23.744 | 76.13 | 111.96 | -88.1 |
| Western Europe | -1630.16 | 1171.629 | 528.176 | -3329.97 | -71.87 | -32.4 | 204.27 |
| Western Sub-Saharan Africa | 167.66 | -108.815 | 575.202 | -298.729 | -64.9 | 343.08 | -178.18 |
| Eastern Europe | -281.01 | 94.816 | -7.128 | -368.695 | -33.74 | 2.54 | 131.2 |
| North Africa and Middle East | 167.62 | 80.717 | 302.259 | -215.352 | 48.15 | 180.32 | -128.48 |
| Australasia | 144.94 | 46.393 | 46.924 | 51.625 | 32.01 | 32.37 | 35.62 |
| Caribbean | -0.69 | 4.779 | 4.972 | -10.439 | -692.61 | -720.58 | 1512.9 |
| Oceania | 2.01 | 0.2 | 1.009 | 0.798 | 9.95 | 50.2 | 39.7 |
